# The interplay between noncoding RNA and YAP/TAZ signaling in cancers: molecular functions and mechanisms

**DOI:** 10.1186/s13046-022-02403-4

**Published:** 2022-06-14

**Authors:** Yirao Zhang, Yang Wang, Hao Ji, Jie Ding, Keming Wang

**Affiliations:** 1 Department of Oncology, Second Affiliated Hospital of Nanjing Medical University, Nanjing, 210011 Jiangsu China; 2 Department of Liver Surgery and Liver Transplantation Center, School of Medicine, Renji Hospital, Shanghai Jiao Tong University, Shanghai, 200127 China

**Keywords:** YAP, TAZ, ncRNA, Carcinogenesis

## Abstract

The Hippo signaling pathway was found coordinately modulates cell regeneration and organ size. Its dysregulation contributes to uncontrolled cell proliferation and malignant transformation. YAP/TAZ are two critical effectors of the Hippo pathway and have been demonstrated essential for the initiation or growth of most tumors. Noncoding RNAs (ncRNAs), including miRNAs, lncRNAs, and circRNAs, have been shown to play critical roles in the development of many cancers. In the past few decades, a growing number of studies have revealed that ncRNAs can directly or indirectly regulate YAP/TAZ signaling. YAP/TAZ also regulate ncRNAs expression in return. This review summarizes the interactions between YAP/TAZ signaling and noncoding RNAs together with their biological functions on cancer progression. We also try to describe the complex feedback loop existing between these components.

## Background

The Hippo signaling pathway was initially discovered in Drosophila and regulates organ growth and cell plasticity during animal development and regeneration [1]. The transcriptional coactivators Yes-associated protein (YAP) and transcriptional coactivator with PDZ-binding motif (TAZ) are two key effectors that mediate the major gene regulation and biological functions of the Hippo pathway [2]. Importantly, YAP/TAZ exert physiological functions dependent on their transfer from the cytoplasm to the nucleus. Nuclear YAP/TAZ interact with transcription factors, particularly TEA domain (TEAD) family members, to induce the expression of proproliferative and survival-enhancing genes. In recent years, studies have found that YAP/TAZ are hyperactivated in human cancers and that excessive activation of YAP/TAZ correlates with poor outcome in several cancer types [3–6]. YAP/TAZ, as oncogenes, are known to be involved in cancer cell proliferation, invasion, and metastasis; epithelial-mesenchymal transition (EMT); and resistance to anticancer drugs [7]. Treating tumor cells with YAP/TAZ inhibitors, such as verteporfin, significantly reversed the malignant biological behavior of the tumor cells [8–11], indicating the therapeutic value of targeting YAP/TAZ signaling in cancer. Notably, an increasing number of studies have reported the effects of YAP/TAZ in the tumor microenvironment, which further emphasizes the essential role of YAP/TAZ in tumorigenesis. Therefore, understanding the regulation and mechanisms of YAP/TAZ signaling in cancer is of great importance.

Noncoding RNAs (ncRNAs) are transcripts that do not encode proteins and are classified as microRNAs (miRNAs), circular RNAs (circRNAs), and long noncoding RNAs (lncRNAs). Although ncRNAs lack protein-coding capacity, subsequent studies have shown that ncRNAs are key regulators mediating many fundamental cellular processes, such as differentiation, proliferation, apoptosis, and cell metabolism. NcRNAs can act as archetypical decoys, signals, guides, and scaffolds to mediate mRNA transcription and translation, protein abundance and localization, and chromatin and protein conformation [12–15]. Additionally, a limited number of ncRNAs that can encode peptides with biological and pathological functions have been validated [16]. A large body of evidence has demonstrated that ncRNAs are important versatile molecules involved in various tumorigenic processes. In vivo, ncRNAs are remarkably stable and are thus potential biomarkers for the diagnosis and prognosis of cancer patients [17–21].

The major roles of ncRNAs in YAP/TAZ signaling, such as regulating the transcription, localization, and stability of YAP/TAZ, has been highlighted by recent studies. In turn, some studies have reported that YAP/TAZ are upstream mediators of ncRNAs. In this review, we discuss the crosstalk between YAP/TAZ and ncRNAs and describe their biological functions in cancers. We expect that YAP/TAZ-associated ncRNAs could be molecular targets and prognostic biomarkers in various cancers.

## The Hippo signaling pathway

The core of the classical Hippo pathway comprises two Ser/Thr kinases: Ste20-like kinase 1/2 (Mst1/2) and large tumor suppressor kinase 1/2 (LATS1/2). MST1/2, aided by its partner Salvador1 (SAV1), stimulate LATS1/2 and the LATS cofactor MOB1 [22]. Subsequently, the serine/threonine-protein kinase LATS1 directly phosphorylates YAP and TAZ, facilitating their cytoplasmic localization or degradation. Indeed, LATS-mediated phosphorylation at YAP Ser127 generates a unique 14–3-3 protein binding site leading to YAP cytoplasmic localization [23]. YAP is phosphorylated not only at Ser127 but also at Ser381 by LATS1/2, which primes YAP for subsequent phosphorylation by casein kinase 1 (CK1) delta/epsilon in a phosphodegron. The phosphorylated phosphodegron then recruits the β-TrCP E3 ubiquitin ligase, which catalyzes the ubiquitination of YAP, ultimately leading to its proteasomal degradation [24]. Once the Hippo pathway is inactivated, YAP/TAZ accumulate in the nucleus and bind to transcription factors such as TEAD1-4/TEF, p73, RUNX, or SMAD to promote the expression of downstream genes [25].

Continuously accumulating research has identified additional regulators that should be considered novel members of the Hippo core cassette. For instance, Ras association domain family protein 1 isoform A (RASSF1A), a critical upstream regulator of the core Hippo pathway, binds to MST1/2 kinases and SAV1 to activate kinase cascade signaling, leading to YAP/TAZ phosphorylation and inhibition [26]. As another example, nuclear Dbf2-related 1/2 (NDR1/2) kinases, which share a similar NDR domain with LATS1/2 and are phosphorylated by MST1/2 and MOB1, can function as components in an extended Hippo pathway. Active MST1/2 can inhibit YAP/TAZ signaling through NDR1/2-mediated phosphorylation of YAP/TAZ [27]. Notably, Nemo-like kinase (NLK) can phosphorylate YAP at Ser128, blocking its interaction with 14–3-3 and enhancing its nuclear localization [28, 29]. Furthermore, actin-like 6A (ACTL6A) was found to physically interact with YAP/TAZ and disrupt the interaction between YAP and the E3 ubiquitin ligase β-TrCP, thus protecting the YAP protein from degradation [30]. In addition, AJUBA is a multiple LIM domain-containing protein that tends to interact with a large number of proteins. AJUBA has been shown to physically interact with LATS1/2 and inhibit YAP phosphorylation via sequestration of LATS1/2 [31].

**Fig. 1:**
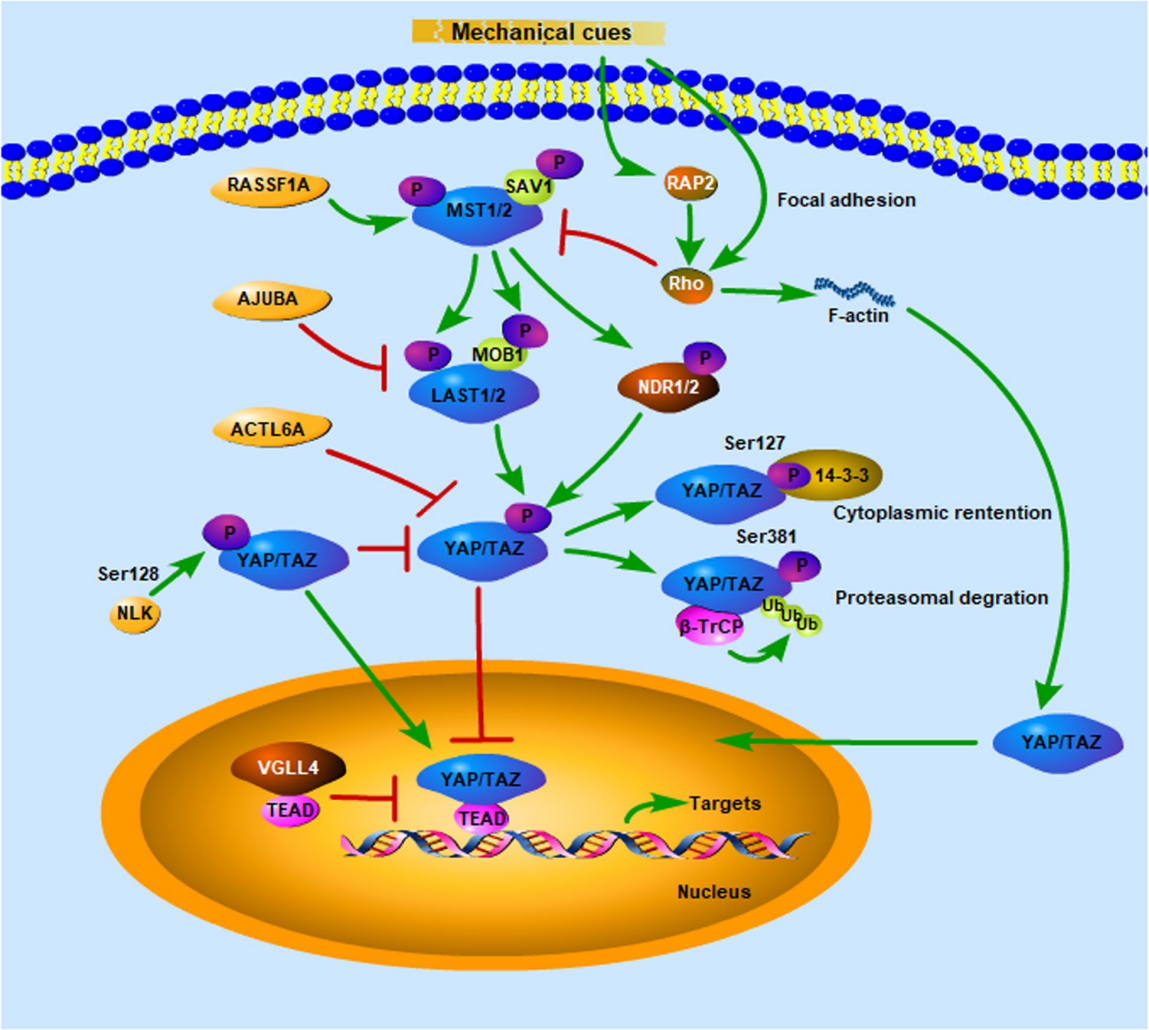
Pathway crosstalk regulates YAP and TAZ YAP/TAZ signaling is mainly regulated by the Hippo signaling pathway. Activation of the Hippo pathway is associated with phosphorylation of the core Hippo kinases MST1/2 and LATS1/2, and their cofactors SAV1 and MOB. Phosphorylated YAP/TAZ are exported from the nucleus and degraded in the cytoplasm. When these kinases are inactive, YAP/TAZ accumulate in the nucleus, bind to transcription factors, and promote the expression of target genes. Additional regulators that compose the Hippo pathway have been identified. Moreover, YAP/TAZ signaling is related to mechanotransduction pathways and is regulated by mechanical forces through the actin cytoskeleton

YAP/TAZ can also be regulated by other mechanisms in addition to the Hippo signaling pathway (Fig. 1). It has long been appreciated that cell behavior can be influenced by mechanical cues such as the stiffness of the ECM. YAP/TAZ have been acknowledged to be critical mediators of mechanotransduction pathways. Dupont found that YAP/TAZ were nuclear and active in cells grown on a stiff extracellular matrix (ECM), whereas they were predominantly cytoplasmic and inactive in cells grown on a soft ECM. These mechanical cues affect YAP/TAZ nuclear localization and activity through a cytoskeletal pathway that requires Rho, a small GTPase that regulates the formation of actin bundles and stress fibers [32]. A recent study reported that low ECM stiffness induced YAP/TAZ cytoplasmic localization by activating MST1/2 via the small GTPase RAP2, suggesting the involvement of both Hippo-dependent and Hippo-independent mechanisms in mechanoregulated YAP/TAZ signaling [33].

## The function of YAP/TAZ in cancer

Extensive research has demonstrated that YAP/TAZ are involved in cancer cell proliferation, metastasis and invasion; EMT; the acquisition of cancer stem cell (CSC) properties; and drug resistance [34–36]. In fact, YAP/TAZ have been identified as signaling hubs in complex networks of various oncogenic signaling pathways such as the EGFR, TGFβ, Wnt, PI3K, GPCR, and KRAS pathways, many of which are considered to be hallmarks of cancer [37, 38]. YAP/TAZ are often also involved in metabolic regulation, such as the promotion of glycolysis, lipogenesis, and glutaminolysis, suggesting that YAP/TAZ are emerging nodes in the provision of energy and necessary synthetic materials for the activities of tumor cells. In addition, YAP/TAZ can regulate the production of secretory proteins, such as amphiregulin (AREG; an epidermal growth factor (EGF) family member), cysteine-rich angiogenic inducer 61 (CYR61), and connective tissue growth factor (CTGF), to influence the tumor microenvironment [39]. Several recent studies have shown that YAP/TAZ mediate the expression of PD-L1, which is involved in cancer immune evasion [40].

Conferring cancer cell stemness is probably one of the best characterized YAP/TAZ-dependent biological responses. CSCs are cancer cells that possess the same self-renewal and differentiation capacities as stem cells, thereby maintaining the ability to regenerate a heterogeneous tumor mass [41]. Recent studies have suggested that CSCs are responsible for tumor initiation, progression, and therapeutic resistance. YAP/TAZ activation can endow cell plasticity by reprogramming genetically normal primary differentiated cells into the corresponding tissue-specific stem or progenitor cells [42]. Many studies have reported that active YAP and TAZ convert benign neoplastic cells into cancer stem cells, contributing to the uncontrolled proliferation, survival, chemoresistance, and metastasis of cancer cells [43–45].

Together, these studies reveal the oncogenic role of YAP/TAZ signaling and provide evidence for the therapeutic benefits of YAP/TAZ inhibition in cancer. Therefore, we attempt to provide an overview of different options that can be utilized to target YAP/TAZ for therapeutic interventions in cancer.

## The crosstalk between YAP/TAZ and microRNAs

### Regulation of YAP/TAZ by microRNAs

MiRNAs are well-known small ncRNAs of 20 to 24 nucleotides in length that regulate the expression of target RNAs through imperfect base pairing with the 3’-untranslated region (3’-UTR) of the target RNAs. A large number of YAP/TAZ-related miRNAs have been discovered in cancer. MiRNAs appear to regulate the YAP/TAZ signaling pathway by targeting YAP/TAZ directly or in a Hippo pathway-dependent manner. Table 1 lists some miRNAs regulating YAP/TAZ expression in cancers.

**Table 1:** miRNAs regulate the expression of YAP/TAZ and cancer progression

MiRNAs	Cancer type	Role in cancer	Targets	Functions	Reference
miR-599	bladder urothelial carcinoma	tumor suppressor	YAP1	inhibit proliferation, invasion, promote apoptosis	[46]
miR-15a-5p	cervical cancer	tumor suppressor	YAP1	inhibit viability, migration and invasion, induce apoptosis	[47]
miR-591	breast cancer	tumor suppressor	YAP1	inhibit proliferation and invasion	[48]
miR-1285-3p	osteosarcoma	tumor suppressor	YAP1	inhibit proliferation and invasion	[49]
miR-345	non-small cell lung cancer	tumor suppressor	YAP1	inhibit migration and invasion	[50]
miR-424-3p	non-small cell lung cancer	tumor suppressor	YAP1	inhibit proliferation,migration and invasion,enhance chemoresistance	[51]
miR-27a-3p	oral squamous cell carcinoma	tumor suppressor	YAP1	inhibit EMT process	[52]
miR-26b	non-small cell lung cancer	tumor suppressor	TAZ	suppress the cisplatin resistance	[53]
miR-92a-3p	cervical cancer	oncogene	LAST1	promote proliferation,invasion and cell cycle transition	[54]
miR-92	ovarian cancer	oncogene	LATS2	promote proliferation and migration, induce immune suppression	[55]
miRNA-224-3p	retinoblastoma	oncogene	LATS2	promote proliferation and angiogenesis,inhibit apoposis	[56]
miR-31	esophageal squamous cell carcinoma	oncogene	LATS2	promote proliferation, migration, invasion and EMT process, induce apoptosis	[57]
miR-520b	breast cancer	oncogene	LATS2	promote migration, EMT process and cancer cell stemness	[58]
miR-216a-3p	cervical cancer	tumor suppressor	ACTL6A	inhibit proliferation,migration	[59]

### MicroRNAs regulate YAP/TAZ by directly targeting YAP/TAZ mRNA

Mature miRNAs bind to the 3-’UTRs of YAP/TAZ, resulting in their translational inhibition or mRNA degradation. This is the case for miR-550a-3-5p, which is a tumor suppressor targeting YAP directly in various cancers, including oral squamous cell carcinoma, colon cancer, and melanoma. As elucidated by Cao et al., miR-550a-3-5p directly targets YAP and then reduces YAP/TEAD-mediated C–C motif chemokine ligand 2 (CCL2) expression in HPV-positive oral squamous cell carcinoma cells [60]. Upon downregulation of CCL2, miR-550a-3-5p inhibited M2 macrophage polarization, leading to suppression of migration, invasion, and EMT in HPV-positive oral squamous cell carcinoma. Similar phenomena occur in vemurafenib-resistant colon cancer and melanoma cells. MiR-550a-3-5p was found to directly target YAP to inhibit its expression, and overexpression of miR-550a-3-5p sensitized vemurafenib-resistant colon cancer and melanoma cells to vemurafenib [61]. It is suggested that miRNA-mediated YAP inhibition may be a new strategy for cancer treatment. In the next sections, we summarize miRNAs that target YAP/TAZ directly in different cancers.

In breast cancer, miR-27b-3 was found to induce apoptosis while attenuating cancer cell metastasis and invasion. It was reported that miR-27b-3 bound to the 3′-UTR of YAP to reduce its expression. Furthermore, the researchers showed that the local anesthetic ropivacaine suppressed breast cancer progression by regulating the miR-27b-3p/YAP axis[62]. Intriguingly, the association between miR-205 and YAP1 has been explored in activated cancer-associated fibroblasts (CAFs) of breast tumors. Enhancing miRNA-205 expression in CAFs significantly inhibited the angiogenic process via downregulation of YAP1 [63]. MiRNAs and YAP/TAZ play significant roles in proliferation, metastasis, and therapy response of breast cancer.

Increasing evidence suggests that YAP/TAZ signaling activation favors hepatocellular carcinoma progression. Higashi et al. reported that miR-9-3p functioned as a tumor suppressor that inhibited hepatocellular carcinoma cell proliferation by targeting TAZ [64]. As mentioned above, YAP/TAZ signaling can control chemoresistance in cancers. Chen et al. identified YAP1, which was directly targeted by miR-590-5p, as the major dysregulated molecule in adriamycin-resistant hepatocellular carcinoma cells. Their findings further strengthened the evidence that miR-590-5p significantly inhibited the expression of the stem cell markers OCT4, SOX-2, Notch-1, Nanog, and Nestin to endow chemotherapeutic resistance in hepatocellular carcinoma [65]. Similar to miR-590-5p, miR-375 was found to be downregulated in cisplatin-resistant liver cancer cells and also suppressed cisplatin-induced resistance in liver cancer cells [66]. Downregulation of miR-375 activated YAP signaling, resulting in increases in IL-6 and TGF-β expression.

The miRNA/YAP axis has been shown to be a potential therapeutic target in lung cancer. In lung cancer tissue specimens and cell lines, miR-381 was found to be significantly downregulated, which decreased the expression of its direct target YAP. Enhancing miR-381 expression inhibited non-small-cell lung cancer (NSCLC) cell proliferation, invasion, migration, and EMT. In addition, metformin inhibited lung cancer cell growth and metastasis via the miR-381/YAP axis [67]. MiR-7 is another tumor suppressor and reverses gefitinib resistance by directly targeting YAP in NSCLC cells. Notably, miR-7 was downregulated in serum exosomes from patients with lung carcinoma compared with healthy controls, and miR-7 expression was negatively correlated with the response to gefitinib treatment in lung carcinoma patients as well as to their overall survival [68].

MiR-195-5p is a tumor suppressor in colon cancer, inhibiting colon cancer cell proliferation, migration, invasion, and EMT [69]. MiR-195-5p can bind to the 3’-untranslated region (3’-UTR) of YAP1 mRNA and downregulate its expression. Another miRNA targeting YAP directly, miR-375, has been identified in colorectal cancer. MiR-375 was found to be downregulated in colorectal cancer cell lines and tissues. Overexpression of miR-375 derepressed the expression of YAP and partially reversed resistance to 5-fluorouracil in colorectal cancer cells. The miR-375/YAP axis regulates apoptosis and cell cycle regulators, including cyclin D1 and BIRC5, as well as CTGF, a downstream effector of the Hippo pathway [70].

On the other hand, miR-375 was found to be inversely correlated with EMT signatures in prostate cancer clinical samples. The EMT process is a reversible biological process whereby epithelial cells transition to a mesenchymal phenotype. The reverse process is called mesenchymal-epithelial transition (MET). Indeed, miR-375 inhibited the invasion and migration of prostate cancer cells by facilitating the MET process. YAP1 was identified as a direct target of miR-375 in prostate cancer, and overexpression of YAP1 abrogated the inhibitory effect of miR-375 on prostate cancer cells [71].

Similar to its effect in colorectal cancer, YAP/TAZ overexpression promotes gastric cancer progression. MiR-15a and miR-16–1 could be considered diagnostic factors for gastric cancer, reducing YAP1 expression to inhibit the proliferation, monolayer colony formation, invasion, and migration of gastric adenocarcinoma cells [72].

### MicroRNAs regulate the stability or nuclear translocation of the YAP/TAZ proteins

Another mechanism by which miRNAs participate in YAP/TAZ regulatory networks is through effects on the stability or nuclear translocation of the YAP/TAZ proteins. However, the shuttling of YAP/TAZ between the cytoplasm and nucleus is mainly achieved in a phosphorylation-dependent manner. Therefore, these interactions rely primarily on the Hippo pathway or other genes, which have indirect effects.

MiR-103a-3p and miR-429 can directly target LATS2 in colorectal cancer, reducing the phosphorylation of YAP and increasing its nuclear localization. Furthermore, the miR-103a-3p/YAP axis was found to promote the expression of hypoxia-inducible factor 1 alpha (HIF1A), consequently enhancing glycolysis and angiogenesis in colorectal cancer cells [73, 74]. RANBP1 functions as a tumor-promoting factor in colorectal cancer, and it disrupts the Hippo pathway to hyperactivate YAP signaling. It was reported that RANBP1 regulates the Hippo pathway by promoting the processing of precursor miRNAs, including the pri-miRNAs of miR-18a, miR-183, and miR-106 [75].

MiR-25 and miR-107 were found to be expressed at high levels in NSCLC cells compared with normal cells. Through binding to the 3’-UTR of LATS2, miR-25 and miR-107 downregulated its expression. Owing to downregulation of LATS2, YAP signaling was activated to promote the growth and metastasis of NSCLC cells [76, 77]. Moreover, miR-135b, which was upregulated in highly invasive NSCLC cells, was found to improve the migration and invasion abilities of NSCLC in vitro and in vivo. A mechanistic study revealed that miR-135b targeted multiple critical components in the Hippo pathway, including LATS2, β-TrCP, and NDR2, to stabilize the TAZ protein [78].

A similar phenomenon occurs in prostate cancer with upregulation of the miR-302/367 cluster, which consists of miR-302a, miR-302b, miR-302c, miR-302d, and miR-367. The miR-302/367 cluster was found to directly target LATS2 to reduce the phosphorylation of YAP and enhance its nuclear translocation. Functionally, the miR-302/367 cluster enhanced the proliferation, sphere formation, and migration of prostate cancer cells and also drove their resistance to androgen ablation [79].

Myosin phosphatase targeting protein 1 (MYPT1), an upstream regulator of the Hippo pathway, can activate the kinase cascade, resulting in YAP/TAZ inhibition. MiR-30b was found to activate YAP signaling by directly targeting MYPT1, leading to enhanced CSC-like properties as well as resistance to platinum-based therapy in ovarian cancer cells [80].

Wiskott–Aldrich syndrome protein-interacting protein family member 1 (WIPF1) was found to protect YAP/TAZ from proteolysis in a Hippo pathway-independent manner. WIPF1 increased the sequestration of the β-catenin adenomatous polyposis coli (APC)-axin-GSK3 destruction complex to the multivesicular body compartment to reduce the capacity of the complex to degrade YAP/TAZ [81]. Pan et al. demonstrated that miR-141 and miR-200c suppress YAP/TAZ expression by repressing the expression of WIPF1 in pancreatic ductal adenocarcinoma cells [82]. The functional role of miR-141 and miR-200c is to inhibit the EMT process and further suppress cell migration and invasion in pancreatic ductal adenocarcinoma.

Exosomes of endosomal origin are nanoscale membrane vesicles (with a size of 30 ~ 100 nm) that coexist with microvesicles and apoptotic bodies in the extracellular microenvironment [83]. Exosomes can transfer various proteins, DNAs, and RNAs to mediate cell communication. MiR-181a, which was found to be highly expressed in papillary thyroid cancer (PTC) cells, could be delivered to human umbilical vein endothelial cells (HUVECs) by hypoxia-induced exosomes. Exosomal miR-181a promoted the proliferation, migration, and angiogenesis of PTC cells. Specifically, miR-181a inhibited Dishevelled binding antagonist of beta catenin 2 (DACT2) by targeting targeted mixed lineage leukemia 3 (MLL3) [84, 85]. In this way, miR-181a reduced the phosphorylation of YAP and activated the YAP-VEGF axis in HUVECs.

Many studies have reported alternative modulators of YAP/TAZ subcellular localization in addition to the 14–3-3 protein, such as Mastermind-like 1/2 (MAML1/2). MAML1/2 interact with YAP/TAZ through a PPxY motif and WW domain to promote the nuclear localization of YAP/TAZ. Kim et al. showed that miR-30c targeted MAML1 and inhibited MAML1 synthesis in cancer cells, leading to reduced YAP/TAZ nuclear localization [86].

### MicroRNAs regulate transcription of YAP/TAZ

In addition to their fundamental interactions with the 3’-UTR of YAP/TAZ or the core components of the Hippo signaling pathway, miRNAs can exert regulatory impacts on transcription factors and affect the transcription of YAP/TAZ.

The cAMP response element-binding (CREB) protein is a CREB/activating transcription factor family member. CREB binds to the promoter of YAP and increases YAP transcriptional output [87]. MiR-1224, functioning as a tumor suppressor, was found to be downregulated in hepatocellular carcinoma and to inhibit the proliferation of hepatocellular carcinoma cells both in vitro and in vivo. By binding to CREB, miR-1224 repressed its transcription and subsequent activation of the YAP signaling pathway in hepatocellular carcinoma cells [88].

RFX5 was identified to bind to the YAP promoter and induce the transcriptional activation of YAP. It was reported that an RNA-binding protein, LIN28, promoted RFX5 mRNA stability. In NSCLC cells, miR-4319 bound to LIN28 and negatively regulated its expression, leading to destabilization of RFX5, which further impeded YAP expression [89]. MiR-4319 thus exerted a tumor-suppressive effect in NSCLC, inhibiting NSCLC cell proliferation and migration while inducing NSCLC cell apoptosis.

### Regulation of microRNAs by YAP/TAZ

Previously, we described the role of miRNAs as upstream mediators of YAP/TAZ in cancer progression. Notably, studies have demonstrated that YAP/TAZ can participate in regulating miRNA expression and result in tumorigenesis.

In general, YAP/TAZ act as coactivators and cooperate with other transcription factors to mediate downstream gene transcription. The study conducted by Ma’s team revealed that TAZ/TEAD can bind to the promoter of miR-224 and facilitate the expression of miR-224. Furthermore, the TAZ/miR-224 axis inhibited the tumor suppressor SMAD4 to promote the proliferation and migration of osteosarcoma cells [90].

Interestingly, researchers have identified YAP and TAZ as regulators of global miRNA biogenesis via modulation of miRNA-processing enzymes, microprocessor, or the Dicer complex [91]. Mori et al. reported that YAP regulates miRNA biogenesis in a cell density-dependent manner. At a low cell density, nuclear YAP binds and sequesters p72, a Microprocessor component, causing widespread miRNA suppression in cells. At a high cell density, YAP is inactivated by exclusion from the cell nucleus, thereby allowing p72 to associate with microprocessor and pri-miRNAs, resulting in enhanced miRNA biogenesis [92]. However, this finding is controversial. Chaulk et al. reached the opposite conclusion that nuclear TAZ/YAP, which are abundant at a low cell density, are required for efficient pre-miRNA processing. Knockdown of TAZ/YAP in low-density cells or density-mediated sequestration of TAZ/YAP into the cytoplasm reduced the level of LIN28. The decreased level of LIN28 led to accumulation of let-7a and let-7b miRNA, and let-7a and let-7b miRNA downregulated Dicer, resulting in defective processing of pre-miRNAs [93]. This research suggested that YAP increases miRNA expression by supporting Dicer-mediated pre-miRNA processing, and this idea was further verified by Zhang’s team. Zhang et al. demonstrated that YAP negatively regulated p21 expression by promoting the expression of the miR-17 family. Upon upregulation of p21, loss of YAP1 inhibited chondrosarcoma cell proliferation and induced senescence in chondrosarcoma cells [94].

MiRNAs are frequently derived from protein-coding gene introns and are located in transcripts coding for multiple products [95]. MiR-25, miR-93, and miR-106b can bind to the 3′-UTR of P21 and inhibit its expression. YAP/TAZ was found to induce the transcription of the MCM7 gene to enhance the expression of its host miRNAs (miR-25, miR-93, and miR-106b) in NSCLC cells, thereby promoting cell proliferation through inhibition of p21 [96].

### The feedback loop between YAP/TAZ and microRNAs

**Fig. 2:**
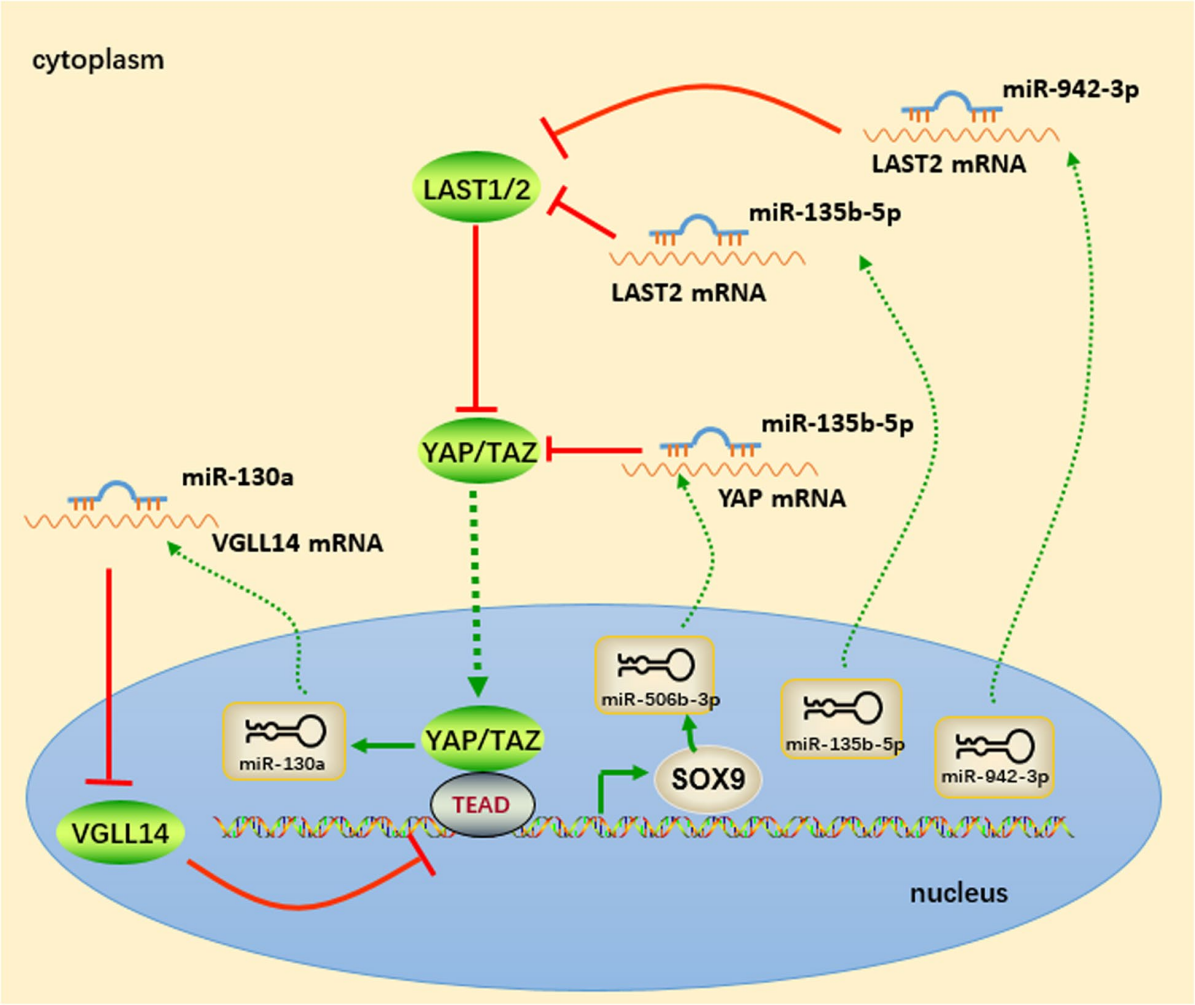
Feedback loops between YAP/TAZ and microRNAs

It is noteworthy that quite a few miRNAs participate in reciprocal feedback loops with the YAP/TAZ signaling pathway (Fig. 2). Shen et al. reported that miR-135b-5p induced by TAZ suppressed glycogen synthase kinase 3β, resulting in EMT induction and promoting the proliferation and invasion of osteosarcoma cells. Moreover, miR-135-5p targeted LATS2 to increase TAZ activity in osteosarcoma cells [97]. The TAZ/miR-135b positive feedback loop played a crucial role in osteosarcoma progression. The positive feedback loop between TAZ and miR-942-3p in bladder cancer is another example. MiR-942-3p was found to be abundantly expressed in bladder cancer, and its upregulation was mediated by TAZ. In turn, overexpression of miR-942-3p amplified TAZ signaling by targeting LATS2, eventually promoting proliferation, angiogenesis, EMT, glycolysis, and reactive oxygen species (ROS) homeostasis in bladder cancer [98].

MiR-19b-3p was suggested to promote lung cancer progression. In detail, exosomal miR-19b-3p was secreted from lung adenocarcinoma cells and taken up by tumor-associated macrophages, leading to the activation of STAT3 signaling. Activated STAT3 signaling induced M2 macrophage polarization and promoted LINC00273 transcription in M2 macrophages. In contrast, M2 macrophages transferred exosomal LINC00273 to lung adenocarcinoma cells. Investigation of molecular pathways demonstrated that LINC00273 recruits NEDD4 to facilitate LATS2 ubiquitination and degradation, leading to the activation of YAP signaling. In turn, activated YAP induced the transcription of RNA-binding motif protein encoded on the X chromosome (RBMX). By binding to miR-19b-3p, RBMX facilitated the packaging of miR-19b-3p into LUAD cell-derived exosomes [99]. Collectively, the miR-19b-3p/LINC00273/YAP feedback loop contributed to the communication between lung cancer cells and tumor-associated macrophages.

It was confirmed that VGLL4 is a YAP signaling inhibitor that competes with YAP for TEAD binding. MiR-130a, which is directly induced by YAP, activated YAP by suppressing VGLL4 expression. YAP signaling was sustained through this miR-130a-dependent positive feedback loop, leading to tumorigenesis and cancer development [100].

## The crosstalk between YAP/TAZ and lncRNAs in cancer

### Regulation of YAP/TAZ by lncRNAs

LncRNAs are a class of noncoding RNAs with a transcript length of more than 200 nucleotides. Aberrant expression of lncRNAs has been reported to contribute to the occurrence and development of multiple cancers. Recently, a continuously increasing number of YAP/TAZ-associated lncRNAs have been discovered. LncRNAs can act as signals, decoys, guides, and scaffolds by interacting with proteins or other RNA molecules. LncRNAs can function as miRNA sponges to prevent the association of miRNAs with downstream mRNAs. Some lncRNAs bind to transcription factors to regulate gene expression. Additionally, lncRNAs can regulate gene expression via histone modification and chromatin remodeling. Table 2 lists some lncRNAs regulating YAP/TAZ expression in cancers.

**Table 2:** lncRNAs regulate the expression of YAP/TAZ and cancer progression

LncRNAs	Cancer type	Role in cancer	Key mechanisms	Functions	Reference
LUADT1	nasopharyngeal carcinoma	oncogene	lncRNA LUADT1 sponges miR-1207-5p to promote YAP/TAZ expression	promote proliferation,invasion and migration	[101]
RP11-51O6.1	colorectal cancer	oncogene	lncRNA RP11-51O6.1 sponges miR-206 to promote YAP1 expression	promote proliferation,migration and invasion	[102]
RP11-757G1.5	colorectal cancer	oncogene	lncRNA RP11-757G1.5 sponges miR-139-5p to promote YAP1 expression	promote proliferation,migration and invasion	[103]
LINC00174	colocrectal carcinoma	oncogene	LINC00174 sponges miR-1910-3p to promote TAZ expression	promote proliferation	[104]
LINC00649	gastric cancer	oncogene	LINC00649 sponges miR-16-5p to promote YAP1 expression	promote proliferation,migartion and EMT process	[105]
HCG18	gastric cancer	oncogene	lncRNA HCG18 sponges miR-141-3p to promote YAP/TAZ expression	promote viability, migration and invasion	[106]
LINC00475	glioma	oncogene	LINC00475 sponges miR-141-3p to promote YAP1 expression	promote proliferation,migration and invasion	[107]
LINC00473	glioma	oncogene	LINC00374 sponges miR-195-5p to promote YAP1 expression	promote proliferation,migration,invasion and inhibit apoptosis	[108]
BCYRN1	glioma	oncogene	lncRNA BCYRN1 sponges miR-125a-5p to promote TAZ expression	promote proliferation,migration and invasion	[109]
KCNQ1OT1	glioma	oncogene	lncRNA KCNQ1OT1 sponges miR-375 to promote YAP expression	promote proliferation,migration, invasion and EMT process	[110]
RBM5-AS1	oral squamous cell carcinoma	oncogene	lncRNA RBM5-AS1 sponges miR-1285-3p to promote YAP1 expression	promote proliferation,migration and invasion	[111]
LEF1-AS1	oral squamous cell carcinoma	oncogene	lncRNA LEF1-AS1 interacts with LAST1 and inhibit the binding of LAST1 with MOB to repress YAP phosphorylation	promote proliferation, migration, induce apoptosis	[112]
PICSAR	cutaneous squamous cell carcinoma	oncogene	lncRNA PICSAR sponges miR-125b to promote YAP1 expression	promote proliferation,invasion and inhibit apoptosis	[113]
DUXAP8	ovarian cancer	oncogene	lncRNA DUXAP8 sponges miR-590-5p to promote YAP1 expression	promote proliferation and inhibit apoptosis	[114]
LINC00857	ovarian cancer	oncogene	LINC00857 sponges miR-486-5p to promote YAP1 expression	promote proliferation,migartion,invasion and glycolysis,inhibit aoptosis	[115]
ASAP1-IT1	ovarian cancer	tumor suppressor	lncRNA ASAP1-IT1 sponge miR 2278 to promote LATS2 expression deactivating YAP1	inhibit proliferation, induce apoptosis	[116]
NOC2L-4.1	cervical cancer	oncogene	lncRNA NOC2L-4.1 sponges miR-630 to promote YAP1 expression	promote proliferation,migration	[117]
APTR	osteosarcoma	oncogene	lncRNA APTR sponges miR-132-3p to promote YAP1 expression	promote proliferation,migration and invasion,inhibit apoptosis	[118]
TUG1	renal cell carcinoma	oncogene	lncRNA TUG1 sponges to miR-9 to promote YAP1 expression	promote proliferation and migration	[119]
CCAT2	laryngeal squamous cell carcinoma	oncogene	lncRNA CCAT2 binds to YAP protein and blocks the phosphorylation of YAP induced by LATS1	promote viablity, invasion	[120]
PWAR6	pancreatic ductal adenocarcinoma	oncogene	lncRNA PWAR6 recruit EZH2 to the promoter of YAP1 to repress YAP1 expression	inhibit proliferation, invasion and migration, induce apoptosis	[121]

### LncRNAs regulate YAP/TAZ signaling by functioning as ceRNAs

The classical mechanism by which lncRNAs participate in YAP/TAZ signaling is through binding to miRNAs as competitive endogenous RNAs (ceRNAs), thereby affecting the expression of YAP/TAZ or various executors in the Hippo pathway.

Yan et al. reported that the lncRNA FLVCR1-AS1 level was elevated in ovarian serous cancer (OSC) tissues and sera. Functionally, FLVCR1-AS1 increased OSC cell proliferation, migration, invasion, and EMT and decreased OSC cell apoptosis in vitro and in vivo. Further study showed that FLVCR1-AS1 activated YAP signaling by acting as a decoy for miR-513 that bound to the 3’ UTR of YAP [122]. Similarly, the lncRNA MLK7-AS1 affects YAP/TAZ signaling in ovarian cancer by regulating the YAP level via the ceRNA mechanism. The lncRNA MLK7-AS1 promoted proliferation, metastasis, and the EMT process via the miR-375/YAP axis in ovarian cancer [123]. Furthermore, the lncRNA MIR205HG was found to deplete endogenous miR-590-3p to increase YAP expression, inducing uncontrolled proliferation of head and neck cancer cells [124]. Additionally, Wu et al. reported that the lncRNA SNHG15 sponged miR-200a-3p to positively modulate YAP1 expression. Knockdown of SNHG15 inhibited proliferation, migration, and EMT and promoted apoptosis in papillary thyroid carcinoma cells [125].

Importantly, lncRNA–miRNA networks can regulate YAP/TAZ signaling by affecting upstream mediators in the Hippo pathway. For example, the lncRNA ASMTL-AS1 sequesters miR-342-3p to enhance the expression of NLK, which can phosphorylate YAP at Ser128, leading to enhanced nuclear translocation of YAP [126]. The lncRNA ASMTL-AS1 was found to be highly expressed in hepatocellular carcinoma tissues, especially in residual hepatocellular carcinoma tissues after insufficient radiofrequency ablation, and its expression level was related to stage and prognosis in hepatocellular carcinoma. Another study revealed the role of the lncRNA MRVI1-AS1 in nasopharyngeal cancer chemosensitivity by inhibiting TAZ activation. Mechanistically, the lncRNA MRVI1-AS1 inhibited the expression of miR-513a-5p and miR-27b-3p by sponging them to upregulate ATF3 (activating transcription factor 3) expression. Furthermore, the MRVI1-AS1/ATF3 axis promoted the expression of RASSF1 (Ras association domain family member 1), a Hippo pathway regulatory factor, thereby inhibiting TAZ expression [127].

Overall, these studies suggest the regulatory roles of lncRNA–miRNA networks in YAP/TAZ signaling in different cancers.

### LncRNAs regulate YAP/TAZ signaling by affecting protein–DNA interactions

Another mechanism by which lncRNAs regulate gene expression is through binding to transcription factors to mediate DNA transcription. By binding to C-Myc at the YAP1 promoter, the lncRNA RP11-323N12.5 promotes YAP1 expression in gastric cancer. Furthermore, the lncRNA RP11-323N12.5 was found to mediate immunosuppression by upregulating YAP-induced immunosuppression-related genes, including PSTG2, CSF1, CXCL15, and CSF3 [128]. LINC01048 is another tumor promoter that enhances proliferation while inhibiting apoptosis in cutaneous squamous cell carcinoma cells by increasing YAP1 expression. Further investigation revealed that LINC01048 recruits TAF15 (a transcriptional activator) to the YAP1 promoter to transcriptionally activate YAP1 in cutaneous squamous cell carcinoma cells [129].

Previously, the AJUBA protein was shown to activate YAP signaling by inhibiting MST1/2 and LATS1. The lncRNA MNX1-AS1, which was found to be elevated in intrahepatic cholangiocarcinoma cell lines and tissues, facilitated proliferation, migration, invasion, and angiogenesis in intrahepatic cholangiocarcinoma [130]. Mechanistically, the lncRNA MNX1-AS1 recruited C-Myc and MAZ to the nucleus to facilitate the expression of MNX1. Then, MNX1 promoted the expression of the AJUBA protein by binding to the AJUBA promoter region. Owing to the increased expression of AJUBA, YAP signaling was activated in intrahepatic cholangiocarcinoma.

### LncRNAs regulate YAP/TAZ signaling by affecting protein–RNA interactions

LncRNAs also play an essential role in posttranscriptional regulation by interacting with RNA-binding proteins. HuR, a nucleocytoplasmic shuttling protein, can regulate the stability of its target mRNAs [131]. The lncRNA B4GALT1-AS1, whose expression was found to be significantly increased in osteosarcoma tissues and cell lines, promoted proliferation, migration, stemness, and adriamycin-therapeutic sensitivity in osteosarcoma. Mechanistically, the lncRNA B4GALT1-AS1 enhanced YAP mRNA stability by recruiting HuR [132].

### LncRNAs regulate YAP/TAZ signaling by affecting protein–chromatin interactions

Chromatin is a macromolecular complex comprising DNA, histone proteins, and RNA [133]. Methylation of histone lysines is a core mechanism that affects gene expression and genome stability. Liu et al. reported that the lncRNA HOTTIP maintained osteosarcoma cell viability, proliferation, migration, and invasion in vitro and contributed to xenograft growth or lung metastasis in vivo. Mechanistically, the lncRNA HOTTIP acted as a scaffold to recruit enhancer of zeste homolog 2 (EZH2) and lysine‐specific demethylase 1 (LSD1) to stimulate histone H3 lysine 27 trimethylation and inhibit histone H3 lysine 4 dimethylation of LATS2. Generally, histone H3 lysine 27 trimethylation contributes to gene silencing, whereas histone H3 lysine 4 dimethylation is associated with gene activation [134, 135]. Via this mechanism, LATS2 was epigenetically silenced, leading to YAP signaling activation [136].

### LncRNAs regulate YAP/TAZ signaling by driving aberrant liquid–liquid phase separation

The formation of biomolecular condensates via liquid–liquid phase separation (LLPS) has recently emerged as a widespread mechanism underlying the spatiotemporal coordination of biological activities in cells [137]. LncRNAs contain repetitive sequences and thus contribute to LLPS [138]. Recently, a study revealed the lncRNA SNHG9 as a tumor-promoting factor in breast cancer, and the expression of SNHG9 was positively correlated with breast cancer progression. Mechanistically, the lncRNA SNHG9 and its associated phosphatidic acids (PA) interact with the C-terminal domain of LATS1, driving liquid droplet formation of LATS1. Furthermore, LATS1 kinase activity was impaired by LLPS promoted by SNHG9 and PA, resulting in reduced YAP phosphorylation and cytoplasmic translocation [139].

### Regulation of lncRNAs by YAP/TAZ

As has been shown for miRNAs, some studies have demonstrated that YAP/TAZ can regulate lncRNAs and result in cancer progression. For instance, the lncRNA H19, an oncogenic mediator in various cancers, was found to be upregulated by YAP in osteosarcoma [140]. Another study showed that highly expressed YAP facilitated the expression of the lncRNA BCAR4, which coordinated Hedgehog signaling to enhance the transcription of the glycolysis activators HK2 and PFKFB3, thus reprogramming glucose metabolism and conferring invasiveness and a propensity for metastasis on triple-negative breast cancer cells [141, 142]. In colorectal cancer, the lncRNA MALAT1 was found to promote angiogenesis and the EMT process by sponging miR-126-5p to increase VEGFA, Slug, and Twist expression, and LINC00958 was positively regulated by YAP1, which promoted the transcription of LINC00958 by forming a complex with β-catenin/transcription factor 4 that bound to the MALAT1 promoter [143].

Although YAP/TAZ are known to function as transcriptional coactivators, recent transcriptomic studies have revealed that they can also suppress gene expression. Their transcriptional repressive function depends on TEADs and the NuRD complex, and the latter promotes histone deacetylation and nucleosome occupancy at target genes[144]. Tan et al. showed that the lncRNA NORAD was downregulated in lung and breast cancers and that low expression of the lncRNA NORAD in these cancer types was associated with lymph node metastasis and poor prognosis. The YAP/TAZ-TEAD-NuRD complex transcriptionally repressed NORAD expression, which contributed to YAP/TEAD-induced tumor migration and invasion [145].

### Feedback loops between lncRNAs and YAP/TAZ

**Fig. 3:**
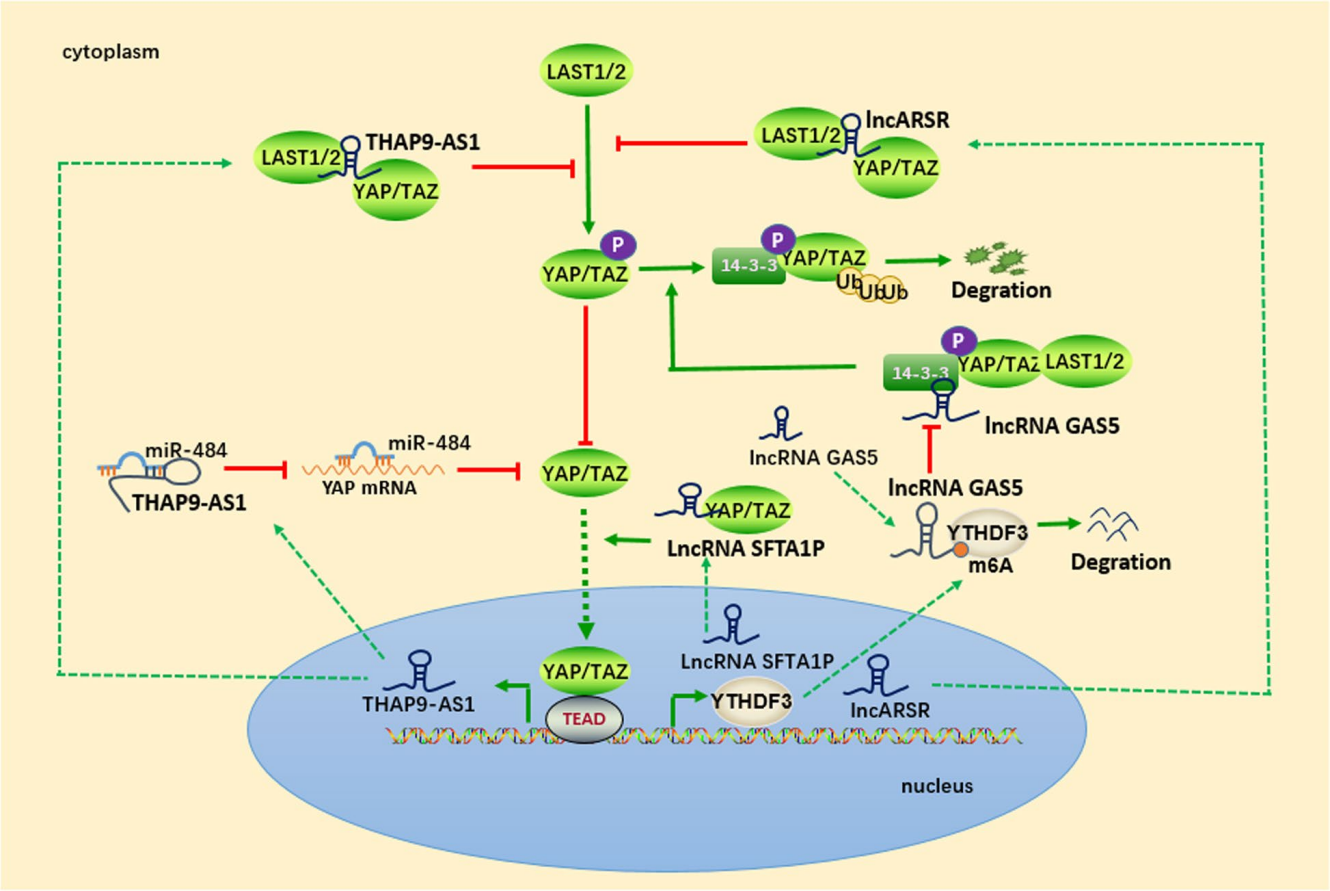
Feedback loops between YAP/TAZ and lncRNAs

Feedback loops between YAP/TAZ and lncRNAs have also received attention (Fig. 3). In primary renal tumor-initiating cells (T-ICs), lncARSR was found to be transcriptionally upregulated by YAP; afterward, the increased lncARSR bound specifically to the WW1/2 domain of YAP and protected YAP from phosphorylation by LATS1, thus forming a positive feedback loop. According to the study by Qu et al., the YAP/lncARSR interaction enhanced the self-renewal capacity, tumorigenicity, and metastasis of renal T-ICs [146]. Similarly, in pancreatic ductal adenocarcinoma cells, the YAP-induced lncRNA THAP9-AS1 stimulated YAP signaling activation by sponging miR-484 and binding to the YAP protein to inhibit LATS1-mediated YAP phosphorylation, eventually enhancing YAP expression at both the posttranscriptional and posttranslational levels [147]. Additionally, a study showed that the lncRNA SFTA1P bound to TAZ to promote its nuclear translocation, resulting in YAP/TAZ signaling activation, in NSCLC cells. Based on the mutually promotive relationship between the lncRNA SFTA1P and TAZ, they were integrated into a positive feedback loop that induced proliferation and inhibited programmed cell death in NSCLC cells [148] Figs. 2 and 3.

Regarding the role of the lncRNA GAS5 as a tumor suppressor in colorectal cancer, Ni et al. uncovered a negative feedback loop of the lncRNA GAS5-YAP-YTHDF3 axis in the progression of colorectal cancer. The lncRNA GAS5 functions as an RNA scaffold binding to the WW domain of YAP to facilitate interactions between YAP and 14–3-3 proteins or LATS1, subsequently increasing the ubiquitin-mediated degradation of YAP. Importantly, the authors showed a novel target of YAP, YTHDF3. The lncRNA GAS5 decreased YAP-mediated transcription of YTHDF3, which reversibly bound to N^6^-methyladenosine (m6A)-methylated GAS5 to trigger its decay [149].

## The crosstalk between circRNAs and YAP/TAZ

### Regulation of YAP/TAZ by circRNAs

Circular RNAs (circRNAs) were first discovered in plant viroids and Sendai virus in 1976 [150]. Unlike linear RNAs, circRNAs have a covalently closed continuous loop structure without a 5′ cap or a 3′ poly(A) tail [151]. These structural characteristics increase the stability of circRNAs in plasma and other body fluids; thus, they are potential diagnostic and prognostic biomarkers in cancer. CircRNAs can modify various biological activities, for example, regulating transcription and degradation and occasionally acting as polypeptide-producing templates [152]. In addition, like lncRNAs, circRNAs can sponge miRNAs. Recently, circRNAs have been found to have the potential to either promote or suppress cancer development in a YAP/TAZ-dependent manner.

### CircRNAs regulate YAP/TAZ by functioning as ceRNAs

In accordance with the mechanism by which lncRNAs function, circRNAs are becoming a novel research hotspot in the ceRNA family. For instance, circ_0001667 upregulation can promote the proliferation and migration of breast cancer cells by regulating the miR-125a-5p/TAZ signaling pathway [153]. CircRNAs can regulate cancer stemness. CircPIP5K1A is an oncogenic factor in osteosarcoma. Mechanistically, circPIP5K1A was found to enhance YAP expression by targeting miR-515-5p. Knockdown of circPIP5K1A inhibited the CSC properties of osteosarcoma cells, while miR-515-5p inhibitor treatment or YAP overexpression reversed this circPIP5K1A knockdown-induced inhibition [154]. In colorectal cancer, circ_0128846 was found to sponge miR-1184 to release the suppression of AJUBA expression, leading to reduced YAP phosphorylation and activated YAP signaling, thereby promoting proliferation, migration, and invasion and inhibiting apoptosis in colorectal cells [155].

Circ_104075 is a tumor-promoting factor that was found to be highly expressed in hepatocellular carcinoma cell lines and tissues and in the serum of hepatocellular carcinoma patients. The study reported that circ_104075 enhanced YAP expression by acting as a sponge for miR-582-3p, thereby promoting hepatocellular carcinoma progression. Interestingly, an N6-methyladenosine (m6A) motif was identified in the 353–357 base pair region of the YAP 3'UTR, and this m6A modification was essential for the interaction between miR-582-3p and the YAP 3'UTR, indicating that m6A modification of mRNA can impact its stability by regulating its interaction with miRNAs [156].

### CircRNAs regulate YAP/TAZ by affecting protein–mRNA interactions

In the translation initiation complex, eukaryotic initiation factor (eIF) 4G binds to Poly(A)-binding protein (PABP), leading to mRNA circularization and enhancing translation. CircYAP, which is derived from the fourth and fifth exons of YAP, was found to negatively regulate YAP expression by suppressing the assembly of the YAP translation initiation machinery in breast cancer cells. CircYAP specifically recognized and bound to YAP mRNA and simultaneously bound to with eIF4G and PABP. These interactions competitively inhibited the interaction of eIF4G and PABP and therefore suppressed the initiation of YAP translation. This study demonstrated the downregulation of circYap expression in breast cancer tissue and indicated that it markedly suppressed the proliferation, migration and colony formation of breast cancer cells [157].

Poly(rC)-binding protein 2 (PCBP2) belongs to the polycytosine-binding protein family, and it plays a crucial role in regulating mRNA stability, protein translation, and protein–protein interactions [158]. Chen et al. reported that PCBP2 could destabilize YAP mRNAs in hepatocellular carcinoma. CircCPSF6, which was upregulated in hepatocellular carcinoma specimens, competitively interacted with PCBP2 to inhibit its binding to YAP1 mRNA, thus attenuating PCBP2-mediated destabilization of YAP1 [159]. Aberrant activation of the circCPSF6-YAP1 axis drove malignancy in hepatocellular carcinoma.

### CircRNAs regulate YAP/TAZ by encoding small peptides

Intriguingly, circRNAs have been reported to encode small peptides to regulate tumor pathogenesis. CircPPP1R12A was found to activate YAP signaling by encoding a conserved 73-aa small peptide, circPPP1R12A-73aa, which reduced the phosphorylation and promoted the nuclear translocation of YAP by inhibiting the activation of MST1/2 and LATS1/2. CircPPP1R12A thus played an essential role in promoting the proliferation, migration, and invasion of colon cancer cells [160].

Table 3 circRNAs that regulate the expression of YAP/TAZ and cancer progression.

**Table 3:** circRNAs regulate the expression of YAP/TAZ and cancer progression

CircRNA	Cancer type	Role in cancer	Key mechanisms	Functions	Reference
Circ_0000511	breast cancer	oncogene	Circ_0000511 sponges miR-326 to promote TAZ expresssion	promote proliferation,migration and invasion,inhibit apoptosis	[161]
Circ_0091074	breast cancer	oncogene	Circ_0091074 sponges miR-1297 to promote TAZ expression	promote proliferation,cell cycle transition and invasion	[162]
CircFAT1	osteosarcoma	oncogene	CircFAT1 sponges miR-375 to promote YAP1 expression	promote proliferation, migration and invasion, inhibit apoptosis	[163]
Circ_0005273	breast cancer	oncogene	Circ_0005273 sponges to promote YAP1 expresssion	promote proliferation and migration	[164]
Circ_0014235	non-small cell lung cancer	oncogene	Circ_0014235 sponges miR-146b-5p to promote YAP expression	promote proliferation and Gefitinib resistance,inhibit apoptosis	[165]
Circ_0085576	clear cell renal cell carcinoma	oncogene	Circ_0085576 sponges miR-498 to promote YAP1 expresssion	promote proliferation,migration and invasion,induce apoptosis	[166]
Circ_0106714	colorectal cancer	tumor supressor	Circ_0106714 sponges miR-942-5p to promote DLG2 expression result in increasing YAP phosphorylation	inhibit proliferation, migration and invasion, induce apoptosis	[167]
Circ_0000140	oral squamous cell carcinoma	tumor suppressor	Circ_0000140 sponges miR-31 to promote LAST2 expression increasing YAP phosphorylation	inhibit proliferation, migration, invasion and EMT process, induce apoptosis	[168]

### Regulation of circRNAs by YAP/TAZ

Most studies have focused on the role of circRNAs in regulating YAP/TAZ signaling; some studies have revealed YAP/TAZ as regulators of circRNAs. As elucidated by Verduci et al., circPVT1 was overexpressed in head and neck squamous tissues compared to matched normal tissues, and circPVT1 expression was increased in tumors carrying mutant p53 proteins. YAP and mut-p53 proteins are able to physically interact; mut-p53 and TEAD are recruited to the same site where YAP binds the circPVT1 promoter to promote circPVT1 transcription [169]. Mut-p53/YAP/TEAD complex-induced overexpression of circPVT1 enhanced the malignant phenotype in head and neck squamous cell carcinoma. Recently, another study reported that YAP can promote the expression of QKI; then, QKI, as a trans-acting RNA-binding factor involved in circRNA biogenesis, can facilitate circNOTCH1 generation. Further exploration demonstrated that circNOTCH1 can compete with NOTCH1 mRNA for METTL14 binding to increase NOTCH1 mRNA stability, eventually promoting tumor growth, in non-small-cell lung cancer [170].

### Potential clinical applications of YAP/TAZ and ncRNAs

Although cancer is the second leading cause of death, there are still limited numbers of reliable diagnostic, prognostic, and predictive biomarkers. NcRNAs, which exist in different intercellular compartments and can also be identified in biological fluids, may be alternative biomarkers because of their characteristics of abundance and conservation. Moreover, it has been reported that some ncRNAs are cell- and tissue specific. As an example, the circ_104075 level was found to be higher in hepatocellular carcinoma patients than in healthy individuals and patients with hepatitis B, hepatitis C, cirrhosis, lung cancer, gastric cancer, colon cancer, or breast cancer, suggesting that elevated expression of circ_104075 is specific to hepatocellular carcinoma. The area under the receiver operating characteristic curve for circ_104075 was 0.973, with a sensitivity of 96.0% and a specificity of 98.3%, indicating that circ_104075 is a promising serum biomarker for the diagnosis of hepatocellular carcinoma [156]. As another example, circ1662, which directly binds to YAP1 to reduce its phosphorylation and promote its nuclear transfer, exhibited significantly higher expression in colorectal cancer tissues than in paired normal tissues. Expression of circ1662 was positively correlated with poor prognosis and tumor depth in colorectal cancer patients [171]. Similarly, the lncRNA SNHG3, a prognostic factor, was found to contribute to cervical cancer metastasis and initiation via association with YAP1 [172].

Exosomes are usually stable and can exist in body fluids, such as blood, saliva, urine, lacrimal fluid, and cerebrospinal fluid [173]. Chen’s team found that the miR-7 level was significantly higher in serum exosomes from healthy controls than in those from patients with lung carcinoma and that high miR-7 expression was associated with a strong response to gefitinib treatment, as well as longer survival times, in lung carcinoma patients [68]. Therefore, exosomal miR-7 may be a feasible blood-based biomarker for the EGFR T79M resistance mutation.

Previously, YAP/TAZ signaling activation was found to be able to lead to uncontrolled cancer cell proliferation, apoptosis evasion, EMT, and stemness. Notably, EMT and CSCs have been demonstrated to contribute to drug resistance, metastasis, and tumor recurrence. However, due to their unstructured nature, YAP/TAZ are difficult to target using small molecules. YAP/TAZ-associated ncRNAs can be targeted by small molecule inhibitors, antisense oligonucleotides, small interfering RNA/short hairpin RNA-mediated RNA interference techniques and masking of the binding motifs required for interactions between ncRNAs and YAP/TAZ [174]. Clustered regularly interspaced short palindromic repeat (CRISPR)/CRISPR-associated nuclease 9 (Cas9) gene editing can also be applied to manipulate ncRNA sequences [175]. Therefore, biological or pharmacological interventions based on ncRNAs related to YAP/TAZ may become a promising new strategy to reverse drug resistance and improve efficacy of chemotherapy in human cancers. For example, Zhan et al. reported that miR-455-3p inhibited cell proliferation and gemcitabine resistance in pancreatic cancer by targeting TAZ, while inhibition of miR-455-3p had the opposite effects [176]. A similar phenomenon was identified in glioma, in which miR-125b resensitized glioma cells to TNF-related apoptosis-inducing ligand (TRAIL) by suppressing TAZ expression [177]. MiR-509-3p and miR-509-3-5p were markedly downregulated in tissues of recurrent ovarian clear cell carcinoma compared with the paired primary cancer tissues. Furthermore, overexpression of miR-509-3p and miR-509-3-5p reversed cisplatin resistance in ovarian clear cell carcinoma by suppressing the expression of YAP1 [178]. These findings suggest that targeting ncRNAs associated with YAP expression can be considered a strategy to reduce chemoresistance and the relapse rate in cancers.

The lncRNA SNHG29 was found to promote the transcription of PD-L1 by activating YAP signaling, therefore facilitating tumor immune suppression. Moreover, Ni et al. reported that simvastatin suppressed lncRNA SNHG29-mediated YAP activation and promoted antitumor immunity by inhibiting PD-L1 expression [179]. This research uncovered simvastatin as a potential immunotherapeutic drug in colorectal cancer.

MSC-derived exosomes are promising natural nanovectors for drug and molecule delivery [180]. Zhang et al. revealed that exosomal circ_100395 released by AMSCs inhibited the malignant transformation of non-small-cell lung carcinoma cells through the miR-141-3p-LATS2 axis [181]. The transfer of circRNA_100395 via AMSC-derived exosomes is a novel therapeutic strategy for non-small-cell lung carcinoma.

Collectively, these findings indicate that the clinical applications of ncRNAs associated with YAP/TAZ signaling include their use as biomarkers for cancer diagnosis and therapeutic interventions in cancer.

## Conclusion

Accumulating evidence indicates that YAP/TAZ and ncRNAs play essential roles in developing human cancers. As there are complex networks contributing to tumorigenesis, it is insufficient to select therapies targeting single genes. Therefore, improving our understanding of the multiple crosstalk mechanisms and potential feedback loops between ncRNAs and YAP/TAZ is particularly necessary. In this review, we described the interplay between YAP/TAZ and ncRNAs, together with their biological functions in cancers. Also, the crosstalks among miRNA, cirRNA and lncRNA can’t be ignored. We also evaluated YAP/TAZ-associated ncRNAs as biomarkers for cancer diagnosis and prognosis and discussed potential clinical therapeutic applications related to the regulatory mechanisms connecting YAP/TAZ and ncRNAs. However, validation experiments based on sufficient clinical samples for predicting prognosis and ensuring safety in the responsive patient population are still challenging for successful implementation of YAP/TAZ-related ncRNAs in the clinic. Overall, the study of the interplay between YAP/TAZ and ncRNAs may open novel research areas for clinical cancer diagnosis and therapy. 

## Data Availability

Not applicable.

## Abbreviations

YAP: Yes-associated protein
TAZ: Transcriptional co-activator with PDZ-binding motif
EMT: Epithelial-mesenchymal transitio: n
ncRNAs: Non-coding RNAs
miRNAs: MicroRNAs
circRNAs: Circular RNAs
lncRNAs: Long non-coding RNAs
Mst1/2: Ste20-like kinase1/2
SAV1: Salvador1
LATS1/2: Large tumour suppressor kinase 1/2
CK1: Casein kinase 1
ACTL6A: Actin-like 6A
NLK: Nemo-like kinase
RASSF1A: Ras association domain family protein1 isoform A
NDR: Nuclear Dbf2-related
PD-L1: Programmed death 1 ligand 1
CSCs: Cancer stem cells
3’-UTR: 3’-Untranslated region
CAF: Cancer associated-fibroblast
NSCLC: Non-small-cell lung cancer
HIF1A: Hypoxia-inducible factor 1 alpha
VEGFA: Vascular endothelial growth factor-A
MYPT1: Myosin phosphatase targeting proteins1
WIPF1: Wiskott–Aldrich syndrome protein interacting protein family member 1
PTC: Papillary thyroid cancer
HUVEC: Human umbilical vein endothelial cell
MLL3: Mixed-lineage leukemia3
DACT2: Dishevelled binding antagonist of beta catenin 2
CCL2: C–C motif chemokine ligand 2
CREB: CAMP response element-binding
MAML1: Mastermind-like 1
ROS: Reactive oxygen species
EZH2: Enhancer of zeste homolog 2
LSD1: Lysine‐specific demethylase 1
RBMX: RNA-binding motif protein encoded on the X chromosome
HMGCR: 3-Hydroxy-3-methylglutaryl-coenzyme A (CoA) reductase
T-Ics: Tumour-initiating cells
LLPS: Liquid–liquid phase separation
AMSC: Adipose-derived mesenchymal stem cell
PABP: Poly(A)-binding protein
PCBP2: Poly(rC)-binding protein 2
MSC: Mesenchymal stem cell
m6A: N6-methyladenosine
eIF: eukaryotic initiation factor
CRISPR: Clustered regularly interspaced short palindromic repeat
Cas9: CRISPR-associated nuclease 9
TRAIL: TNF-related apoptosis-inducing ligand
